# Idiopathic Thrombocytopenia and Neurologic Manifestations in A Young Female Leading to the Diagnosis of Wilson’s Disease

**Published:** 2012

**Authors:** Seyed Mohammad Salar Zaheryany, Reza Bidaki, Nahid Hemmatian Brujeni, Mohammad Rezvani, Mitra Hakim Shooshtari

**Affiliations:** 1 Tehran University of Medical Sciences, Tehran, Iran; 2 MD. Assistant Professor of Psychiatry, Rafsanjan University of Medical Science Department of Psychiatry, Rafsanjan, Iran.; 3 MD. Neurologist, Tehran University of Medical Sciences, Tehran, Iran.; 4 MD. Assistant of child and adolescence Psychiatry, Tehran University of Medical Sciences, Hazrat Ali Asghar Hospital.

**Keywords:** Apraxia, Kayser Fleischer's ring, Thrombocytopenia, Wilson’s disease

## Abstract

We present a 19-year-old patient with hematologic and neurologic manifestations associated with Wilson’s disease. Idiopathic thrombocytopenia was diagnosed in October 2009. Bone marrow aspiration was normal. Gradually her neurologic and psychiatric symptoms emerged, dysarthria, writing apraxia, learning difficulties, emotionalism and eventually dystonia of hands. The serum ceruloplasmin was low, and the Kayser Fleischer's ring was positive. MRI of the brain showed abnormality in the bilateral basal ganglia, brain stem and superior cerebellar peduncles without post-contrast enhancement.

## Introduction

Hepatolenticular degeneration or Wilson’s disease, in which copper accumulates in tissues and manifests as neurological or psychiatric symptoms and liver disease, is an autosomal recessive genetic disorder (1). Prevalence rate of 1 per 30,000 to 40,000 has been quoted (2, 3). Hematologic manifestations are rare and there are a few case reports indicating idiopathic thrombocytopenia as an initial sign of the patient (4, 5). This case report details one such case.

## Case Presentation

A 19-year-old white female student presented with eight-month history of thrombocytopenia accompanied by different neurologic manifestations. The history of present illness returns to the last summer when her writing and speaking became disturbed and her hand-writing became unreadable. With reopening of schools, her problems progressed, learning was affected and dysarthria and dysgraphia developed. The abnormal points in her lab results were anemia and thrombocytopenia. Bone marrow aspiration was performed which was normal. She reported occasional episodes of dysphagia to solid foods. Ferrous sulfate and folic acid were indicated with recommendation for eating more pistachio and “Jegar” (a kind of dish prepared from the liver of sheep). 

After three months, following an episode of influenza, she got severe pain in her right knee that caused abnormal gait and problems in standing up. Her menstruation became irregular. She noticed that her laughing was out of control and loud. She was gradually affected by task-specific dystonia of her right-hand fingers during writing which progressed to dystonia of her left hand. Salivation increased and jaw claudication appeared. She suffered dystonia of hands, involuntary blinking and hemi-facial spasm.

She denied any psychiatric abnormalities such as depression, anxiety, amnesia and sphincteric symptoms except sleep disturbances in the past few months. Her past medical history was unremarkable except for one episode of febrile seizure when she was one-year-old. Her social history defined her as a nonsmoker teenager living in Tehran with normal relationships with others. She suffered from perfectionism and obsession with her studying and frequently repeated the sentences.

*Physical examination findings*

**Figure1:**
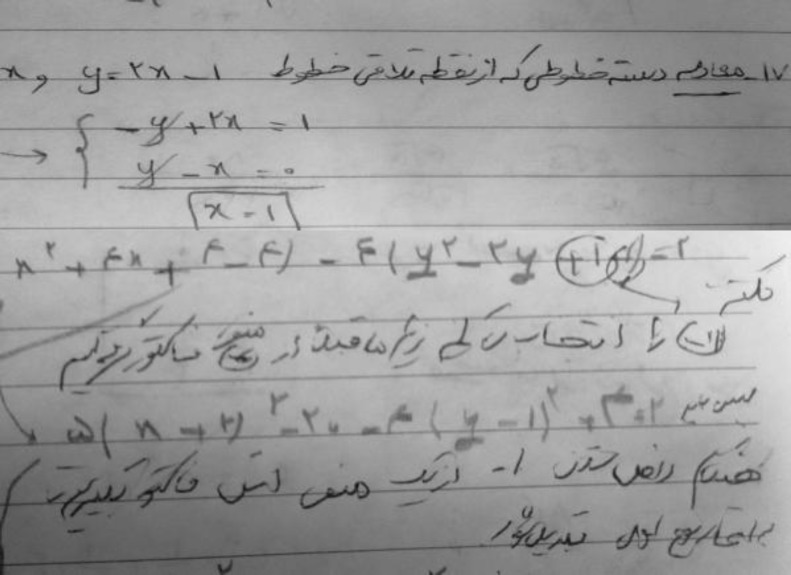
Dysgraphia

Physical examination showed mini mental status examination (MMSE) of 30 scores out of 30. Dysarthric speech and writing apraxia were detected (Figure 1).

**Figure 2:**
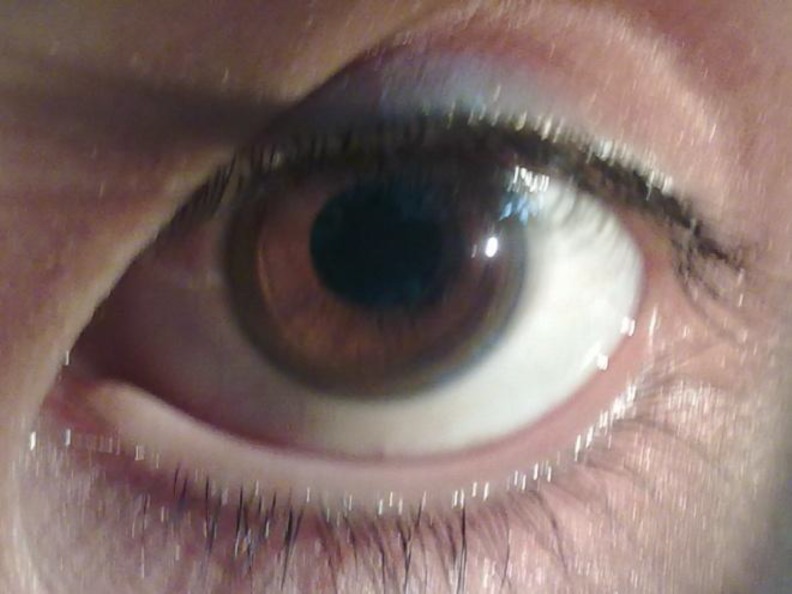
Kayser-Fleischer ring

 Kayser-Fleischer ring was observed and proven by slit lamp examination (Figure 2). 

Eye movements, saccade and pursuit were normal. Other cranial nerves were normal. Spleen was palpable and splenomegaly proved by ultrasonography. Her face showed a state like that of risus sardonicus. Hemifacial spasm, dystonia and choreiform movements of both hands persisted. Bradykinesia and hypokinesia were not detected. Forces and DTRs were normal. Other examinations were normal. 

*Lab Results*

CBC as of 2.5.2010:

WBC: 3.2 × 10^9^/L ↓ 

AST: 46 IU/L↓ 

Total Bill: 1.3 mg/dL 

RBC: 3.7 × 10^12^/L ↓ 

ALT: 33 IU/L 

Total Bill: 1.3 mg/dL↑ 

Hemoglobin: 11.7 gram/dL

BUN: 15 mgr/d 

Direct Bill: 0.4 mg/dL ↑ 

Platelet: 82 × 10^9^/L ↓ 

Cr: 0.9 mgr/dL

APS profile: - ive

Serum ceruloplasmine: 15.6 mg/dL (Ref: 20.39-40.7) ↓

24 Hrs Urine: 

volume 600 ml ,

copper 54 µg (Ref: 15-50) ↑ ,

Cr: 378 mg (Ref: 600-1800) ↓ & Calcium: 75 mg (Ref: 50-300)

*Diagnostic Imaging*

**Figure 3:**
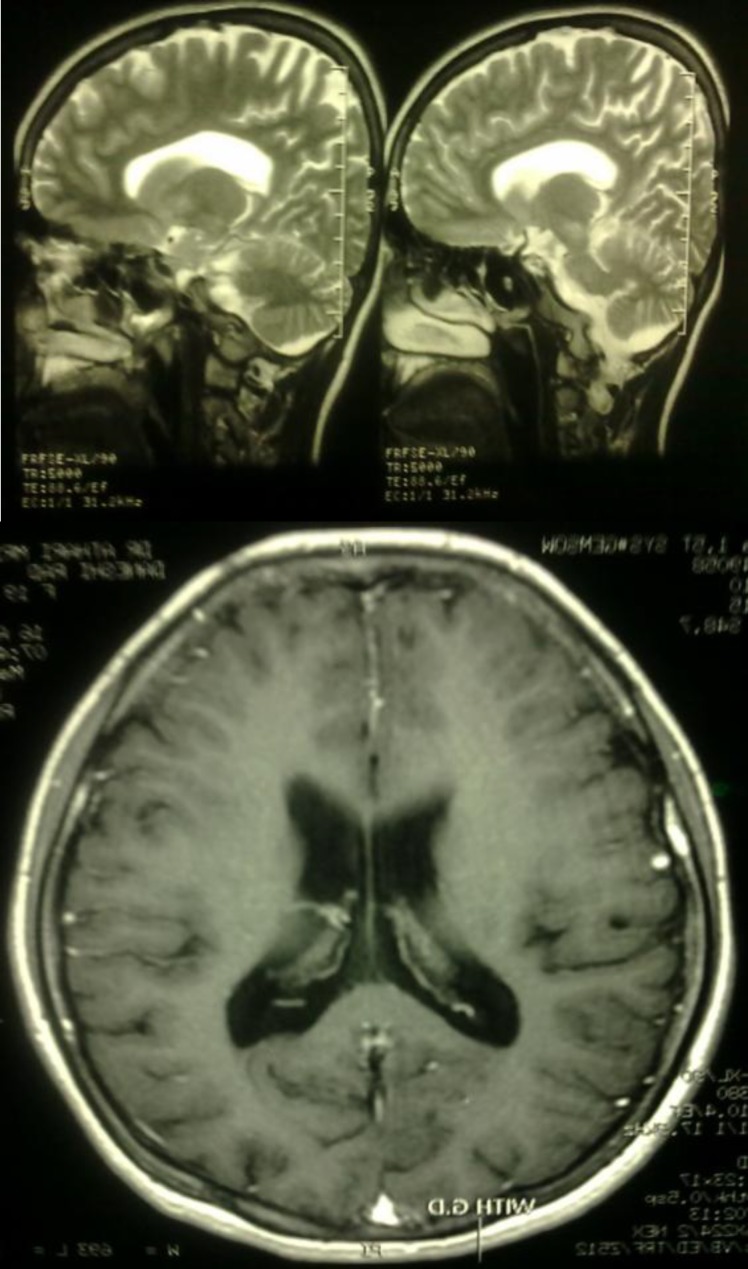
Brain MRI. signal abnormality in the bilateral basal ganglia, brain stem and superior cerebellar peduncles without post-contrast enhancement

Her brain MRI shows signal abnormality in the bilateral basal ganglia, brain stem and superior cerebellar peduncles without post-contrast enhancement (Figure 3).

*Treatment & Follow-up*

D-penicillamine 250 mg/daily, vitamin B6 and prednisolone 20 mg/daily were initiated for the patient. After two days, she got mild fever and aggravation of thrombocytopenia (Platelet: 60×10^9^/L) which after consulting an infectious disease specialist, antibiotic therapy began and her fever improved. Thrombocytopenia developed, therefore her prednisolone was increased to 30 mg/daily and after 3-4 days the number of platelets increased to 80×10^9^/L. Her slurred speech worsened but her hemi-facial spasm and left hand dystonia improved significantly. Blood tests were performed on her brother and were normal. Four months after the treatment, irritability and anxiety had been increased. All neurologic signs and symptoms were been declined. Salivation and spontaneous laughing were been improved.

## Discussion

Of all the causes of here do degenerative dystonia, Wilson’s disease (WD) is the most important because it is treatable, but fatal if left untreated. It is an autosomal recessive disorder of copper metabolism that most commonly presents itself in the first two decades of life. It occurs worldwide with a prevalence of 1–3/100,000 (6).

 Most cases of WD show slurred speech and a movement disorder in adolescence, often in association with behavioral disturbances. Approximately half of all cases present with neuropsychiatric symptoms, usually between 14 and 20 years of age (6).

There is an accumulation of hematologic manifestations such as pancytopenia and neurologic manifestations like slurred speech, writing apraxia and dystonia. An unusual presentation of WD is idiopathic thrombocytopenia which has not been published as of yet in Iran. In a case report published in 1998, an idiopathic thrombocytopenia was presented in the background of WD (4). In another case report published in 2003, a case of WD was presented with idiopathic thrombocytopenic purpura and olfactory paranoid syndrome (5). And in other papers, diagnosis of WD was made according to thrombotic thrombocytopenic purpura-hemolytic uremic syndrome (TTP-HUS) (7, 8).

 Here we should emphasize that bone marrow aspiration was normal as we expect in WD. On the other hand, recommendations for eating more “Jegar”, due to its rich source of copper, aggravated and fastened the progression of the disease.

The ﬁrst neurologic manifestations are most often extrapyramidal with a proclivity to affect the oropharyngeal musculature. The typical presentations are slowness of movement of the tongue, lips, pharynx, larynx, and jaws, resulting in dysarthria, dysphagia, and hoarseness (9). So we can consider dysphagia as well as joint and bone abnormalities symptoms of the disease (6).

The emergence of Parkinsonism, dystonia or a tremor in an adolescent or young adult with slurred speech should always raise the possibility of WD and the combination of a movement disorder with emotionalism, a risus sardonicus and a K-F ring makes the diagnosis highly likely (6).

## Conclusion

Although this case initially presented with idiopathic thrombocytopenia and it is an unusual presentation of WD, but this patient had some important symptoms such as writing apraxia which were neglected and this delayed the diagnosis?

## Authors’contributions

SMSZ participated in treatment and revised the manuscript. RB made the diagnosis and helped to draft the manuscript. SMSZ, NHB, and MR searched and collected the data. MHS helped to edit the manuscript. All authors participated in data analysis, read and approved the final manuscript.

## Notes

**Declaration of interest**: None.
